# Anti-endometriosis Mechanism of *Jiawei Foshou San* Based on Network Pharmacology

**DOI:** 10.3389/fphar.2018.00811

**Published:** 2018-07-26

**Authors:** Yi Chen, Jiahui Wei, Ying Zhang, Wenwei Sun, Zhuoheng Li, Qin Wang, Xiaoyu Xu, Cong Li, Panhong Li

**Affiliations:** ^1^College of Pharmaceutical Sciences and Chinese Medicine, Southwest University, Chongqing, China; ^2^Chongqing Key Laboratory of New Drug Screening from Traditional Chinese Medicine, Chongqing, China; ^3^Pharmacology of Chinese Materia Medica – the Key Discipline Constructed by the State Administration of Traditional Chinese Medicine, Chongqing, China; ^4^Department of Traditional Chinese Medicine and Pharmacy, Chongqing Hospital of Traditional Chinese Medicine, Chongqing, China; ^5^Department of Obstetrics and Gynecology, First Affiliated Hospital of Chongqing Medical University, Chongqing, China

**Keywords:** *Jiawei Foshou San*, endometriosis, network pharmacology, invasion and metastasis, epithelial–mesenchymal transition

## Abstract

*Jiawei Foshou San* (JFS) is the new formula originated from classic *Foshou San* formula, composed with ligustrazine, ferulic acid, and tetrahydropalmatine. Previously JFS inhibited the growth of endometriosis (EMS) with unclear mechanism, especially in metastasis, invasion, and epithelial–mesenchymal transition. In this study, network pharmacology was performed to explore potential mechanism of JFS on EMS. Through compound–compound target and compound target–EMS target networks, key targets were analyzed for pathway enrichment. MMP–TIMP were uncovered as one cluster of the core targets. Furthermore, autologous transplantation of EMS rat’s model were used to evaluate *in vivo* effect of JFS on invasion, metastasis and epithelial–mesenchymal transition. JFS significantly suppressed the growth, and reduced the volume of ectopic endometrium, with modification of pathologic structure. In-depth study, invasion and metastasis were restrained after treating with JFS through decreasing MMP-2 and MMP-9, increasing TIMP-1. Meanwhile, JFS promoted E-cadherin, and attenuated N-cadherin, Vimentin, Snail, Slug, ZEB1, ZEB2, Twist. In brief, anti-EMS effect of JFS might be related to the regulation of epithelial–mesenchymal transformation, thereby inhibition of invasion and metastasis. These findings reveal the potential mechanism of JFS on EMS and the benefit for further evaluation.

## Introduction

Endometriosis is known as the growth of the active endometrial tissue outside the uterus. Even though EMS is considered as a benign gynecological disease, there are malignant performance of invasiveness, angiogenesis, recurrence, and malignant transformation (Ma et al., 2016; Mihailovici et al., 2017). However, the mechanism of EMS is unclear, the reflux theory of menstruation is most widely accepted. It is suggested that invasion and metastasis is a very important step in flowing endometrial tissue (Borrelli et al., 2015; Chui et al., 2017).

In TCM, blood stasis and obstruction of uterus are considered as the main cause of EMS (Shan et al., 2017; Wen et al., 2017). So the treatment of EMS is based on activating blood circulation to dissipate blood stasis (Zhao, 2018). *Foshou San* formula is one of the famous *Huoxue Huayu* recipes, originally reported in *Puji Benshi Fang*. JFS is the new formula originated from classic *Foshou San* formula, composed with ligustrazine, ferulic acid, and tetrahydropalmatine. In previous experiment, good efficiency of JFS has been recovered, including diminishing the growth of EMS, suppression of E_2_, anti-inflammation and anti-angiogenesis (Tang et al., 2014). However, the effect of JFS on invasion, metastasis, and epithelial–mesenchymal transformation has not been reported in EMS.

Traditional Chinese medicine formula has the complicate characteristic of poly-component with poly-target through poly-pathway (Wang et al., 2017). Network pharmacology is a new discipline combined systems biology with drug efficacy, generally describing the connection of multi-component with multi-target and multi-pathway (Ning et al., 2017). So application of network pharmacology on TCM will contribute to illustrate the utility of TCM.

**FIGURE 1:**
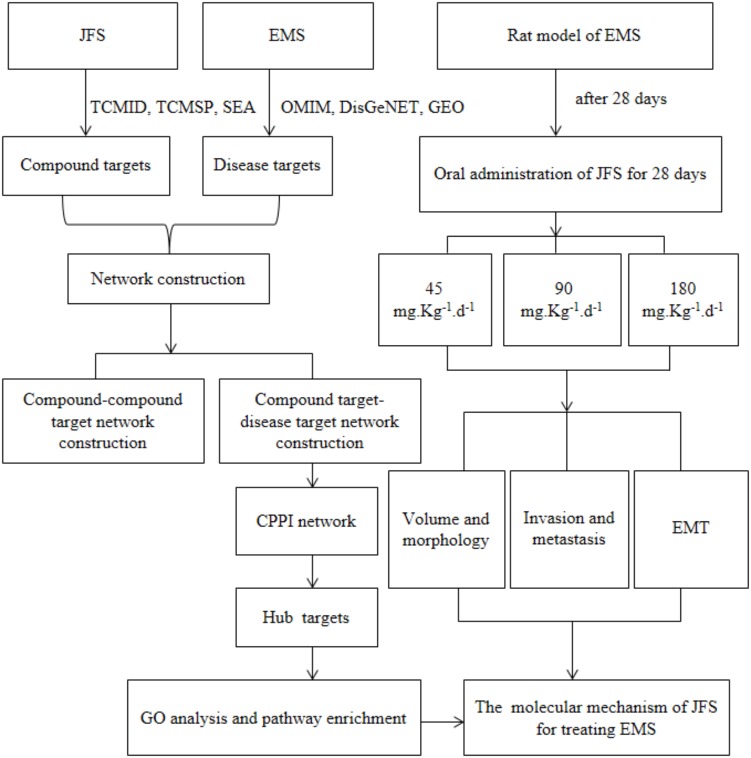
The flowchart of this study based on an integration strategy of network pharmacology and experimental verification. EMS, endometriosis; JFS, *Jiawei Foshou San*; TCMID, Traditional Chinese Medicines Integrated Database; TCMSP, Traditional Chinese Medicine Systems Pharmacology Database and Analysis Platform; SEA, Similarity ensemble approach database; OMIM, Online Mendelian Inheritance in Man; DisGeNET, a database of gene–disease associations; GEO, Gene Expression Omnibus; CPPI, core protein–protein interaction.

In this study, compound–compound target and compound target–EMS target networks were established through network pharmacology data bases. Key targets and pathway enrichment were analyzed. Then autologous transplantation of EMS rat’s model were used to evaluate *in vivo* effect of JFS on EMS. Furthermore, the influence of JFS on invasion, metastasis, and epithelial–mesenchymal transition were investigated (**Figure 1**).

## Materials and Methods

### Collection of Potential Targets for Jiawei Foshou San

In order to collect potential targets of three compounds in JFS as many as possible, the following three databases were used: Traditional Chinese Medicines Integrated Database (TCMID)^1^, Traditional Chinese Medicine Systems Pharmacology Database and Analysis Platform (TCMSP)^2^, and the Similarity ensemble approach (SEA) database^3^.

### Collection of Potential Targets in EMS

The potential targets for EMS were obtained from the three resources using “Endometriosis” as the keyword: Online Mendelian Inheritance in Man (OMIM)^4^, a database of gene–disease associations (DisGeNET)^5^, and Gene Expression Omnibus (GEO)^6^.

### ID Conversion for Searched Targets

All JFS-related and EMS-related targets obtained from databases were aggregated together, and the duplicate ones were removed. Simultaneously, the UniProtKB search function in the protein database UniProt^7^ was used to modify the searched targets to their official names. By entering the target name and limiting the species to “*Homo sapiens*,” multifarious ID types of the targets were converted into UniProt IDs.

### Protein–Protein Interaction Data

The STRING database furnishes both predicted protein–protein interaction information and the data which have been experimentally proven (Szklarczyk et al., 2011). The version 10.5 of STRING^8^ was employed to search for the protein–protein interaction data, with the species limited to “*Homo sapiens*” and a confidence score >0.4 (Tang et al., 2016).

### Network Construction and Analysis

The compound–compound target and compound target–EMS target networks were constructed based on their interaction data and visualized by Cytoscape 3.5.0 software. In the generated networks, nodes represented targets and compounds, edges represented the relationship between them. The targets without interaction were excluded from the network. Afterward, the Network Analyzer, a plugin of Cytoscape, was applied to analyze the topological parameters of each node in the network. Among the topological parameters, degree and betweenness centrality were used as crucial factors to describe the most influential nodes in networks (Li et al., 2015; Tang et al., 2015). Thus, the nodes with higher or equal degrees and betweenness than the average were chosen as the hubs.

### GO Enrichment and Pathway Analysis

The Database for Annotation, Visualization and Integrated Discovery (DAVID)^9^ and the Protein Analysis Through Evolutionary Relationships database (PANTHER)^10^ were applied for Gene Ontology (GO) enrichment and pathway analysis. The specific operation steps were as following, inputting the protein ID and restricting the species to “*Homo sapiens*,” then utilizing the functional annotation tool to make GO enrichment and pathway analysis.

### Animals and Chemicals

Female Sprague-Dawley rats aged 6–7 weeks were purchased from Experimental Animals Institute of Chongqing Academy of Chinese Materia (Certification No: SCXK [yu] 2012-0006). The rats were housed at a *T*a of 20 ± 2°C and 12 h light/dark cycle, free access to food and water in the Experimental Center, College of Pharmaceutical Sciences, Southwest University. This study was carried out in strict accordance with the recommendations in the Guide for the Care and Use of Laboratory Animals of Southwest University (Approval No. 0002183).

Ferulic acid, ligustrazine, and tetrahydropalmatine with the purity of 99.8, 99.3, and 98.1% separately, were provided by Nanjing Zelang Medical Technology Co., Ltd. (Nanjing, China). They were mixed by a ratio of 10:5:3, then suspended in CMC-Na to constitute JFS. Gestrinone was purchased from Zizhu Pharmaceutical Co., Ltd. (Beijing, China).

### Rat Endometriosis Model

Sixty rats with estrus were surgically induced EMS by auto-transplantation of uterine tissue. All operational procedures were conducted as described by Tang et al. (2014) with slight modification. Briefly under sterile condition, uterine horns of anesthetized rats were separated and cut into 5 mm × 5 mm fragments. The uterine segments were suspended in sterile PBS, then sutured onto the inner peritoneum near blood vessels. The incisions were closed and disinfected.

After 28 days of transplantation, the growth of the ectopic endometrium were observed on gross and microscopic examination. The volume of ectopic endometrium were detected by Vernier caliper with volume formula (0.52 × length × width × height) (Pinar et al., 2017). The rats were included with following criteria, viable and well vascularized endometrial explants, and graft volume ≥20 mm^3^ in the second laparotomy.

### Experimental Design

After 28 days of auto-transplantation, EMS model were successfully established in 50 from 60 rats. The success rate of the model was 83%. Those rats were randomly divided into EMS group, low, medium, and high JFS groups and gestrinone group. There were no significant difference in endometriotic volume of each group before treatment (**Table 3**). Another 10 normal female rats were treated as control group without transplantation. 0.5% CMC-Na were administered in control and EMS group. Low, medium, and high JFS groups were given with 45, 90, and 180 mg.kg^-1^.d^-1^ JFS, respectively. A 50 mg.kg^-1^.d^-1^ gestrinone was given in gestrinone group. All the above groups were administered consecutive 28 days by gavage.

### Hematoxylin and Eosin Staining

Eutopic endometrium in control group and ectopic endometrium in other groups were collected and fixed in paraformaldehyde in the end of administration. Then sections from different groups were stained with hematoxylin and eosin (H&E). Endometrial glands and stroma were identified as the essential criteria for diagnosis. The morphological structure were examined and photographed under a microscope (DFC310 FX, Leica, Germany).

### RNA Isolation and Real-Time PCR

The process for RNA isolation and real-time PCR were performed as described previously (Chen et al., 2012). Briefly, TRIzol reagent (Invitrogen, CA, United States) were used for mRNA extraction from the tissues with acid phenol extraction. RT-PCR was carried out using a PrimeScript^TM^ RT reagent Kit (Takara, China) according to the manufacturer’s protocols. Real-time PCR was performed with CFX96 Real-Time System (Bio-Rad, United States) with SYBR^TM^ Green Master Mix (Thermo Fisher Scientific, United States). Rat specific primers were synthesized by Dingguo Changsheng Biotechnology (Beijing, China) (**Table 1**). Each PCR was carried out with the following conditions: 95°C for 2 min, 40 cycles of 95°C for 15 s and 60°C for 1 min. Melt curves were analyzed at the end of each assay to confirm the specificity. Fold change was determined using the 2^-ΔΔCT^ method normalized with endogenous control GAPDH.

**Table 1:** qPCR primer sequence.

Genes		5′–3′
MMP-2	Forward	GGCCCTGTCACTCCTGAGAT
	Reverse	GGCATCCAGGTTATCGGGGA
MMP-9	Forward	TGGACGATGCCTGCAACGTG
	Reverse	GTCGTGCGTGTCCAAAGGCA
TIMP-1	Forward	CAATTCCGACCTCGTCATCAG
	Reverse	CTTGGAACCCTTTATACATCTTGG
E-cadherin	Forward	CTGGACCGAGAGAGTTACCC
	Reverse	GGCACCGACCTCATTCTCAA
N-cadherin	Forward	GCTTCTGGCGGCCTTGCTTCA
	Reverse	GCGTACACTGTGCCGTCCTCATCC
Vimentin	Forward	CCTTGACATTGAGATTGCCA
	Reverse	GTATCAACCAGAGGGAGTGA
Snail	Forward	TTACCTTCCAGCAGCCCTAC
	Reverse	GCTTCGGATGTGCATCTTG
Slug	Forward	ATCTGACCCGTCGACG
	Reverse	CGTCACGACGGGTCAGAT
ZEB1	Forward	GATGGGGCTGCGGATGAG
	Reverse	GCAGGGTGCTCTGGGTCATA
ZEB2	Forward	TCTGCGACATAAATACGA
	Reverse	GAGTGAAGCCTTGAGTGC
Twist	Forward	ACCCTCACACCTCTGCATTC
	Reverse	CAGTTTGATCCCAGCGTTTT
GAPDH	Forward	AGACAGCCGCATCTTCTTGT
	Reverse	CTTGCCGTGGGTAGAGTCAT

### Western Blot Analysis

The protein were separated and extracted from eutopic endometrium in control group and ectopic endometrium in other groups. The tissue lysates were prepared as described previously (Chen et al., 2015, 2017). Briefly quantified protein lysates were separated by SDS-PAGE, transferred to polyvinylidene difluoride membrane (Millipore, United States) and probed with primary rabbit anti-MMP-2, rabbit anti-MMP-9 (1:100 dilution; Boster Biological Technology, Wuhan, China), rabbit anti-TIMP-1 (1:300 dilution; Proteintech Biotechnology, Wuhan, China), rabbit anti-E-cadherin, rabbit anti-Vimentin, rabbit anti-Snail, rabbit anti-Slug (1:1000 dilution; Cell Signaling Technology, Beverly, MA, United States), rabbit anti-β-actin (1:5000 dilution; Proteintech Biotechnology, Wuhan, China) overnight at 4°C. Then the membranes were blotted with an appropriate horseradish peroxidase-linked goat secondary antibody (1:2000 dilution; Zhongshan Golden Bridge Biotechnology, Beijing, China). Electrochemiluminescence was performed according to the manufacturer’s instructions with Tanon 5200 imaging system (Tanon, China). β-Actin was used as endogenous control.

### Statistical Analysis

Data were represented as the arithmetic mean ± SD and compared by one-way ANOVA test using SPSS software (Version 21). *P* < 0.05 was considered statistically significant.

## Results

### Compound–Compound Target Network Construction and Analysis

**FIGURE 2:**
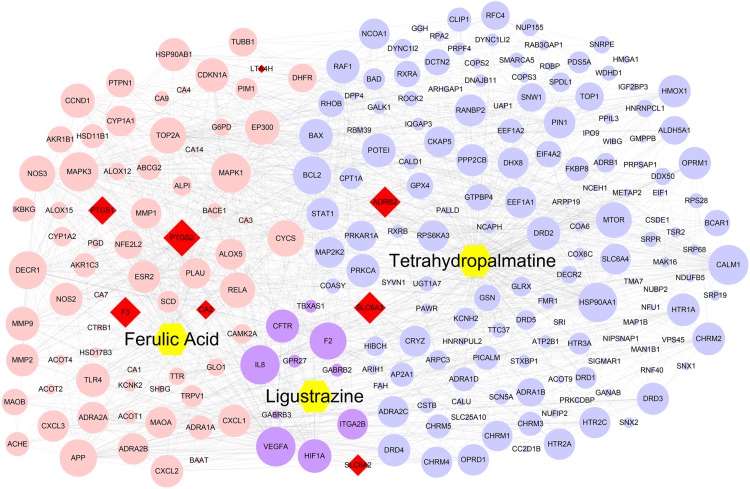
The compound–compound target network. Purple circles, pink circles, blue circles, and red diamonds represented the targets of ligustrazine, ferulic acid, tetrahydropalmatine, and the common targets for all three components, respectively. Yellow hexagons represented the three compounds of *Jiawei Foshou San*. The size of circles and diamonds represented nodes degree value.

A total of 275 potential targets were obtained for three JFS compounds, 10 for ligustrazine, 86 for ferulic acid, and 179 for tetrahydropalmatine. Detailed information is described in **Supplementary Table S1**. The compound–compound target network consisted of 235 nodes and 1508 edges. In this network, most targets belonged to a single compound, while the targets, such as ADRB2, CA2, F3, LTA4H, PTGS1, PTGS2, SLC6A2, and SLC6A3, belonged to more than one compound (**Figure 2**). It was suggested that these uniform targets might be the foundation of synergistic therapeutic effect of TCM.

### Compound Target–Disease Target Network Construction and Analysis

**FIGURE 3:**
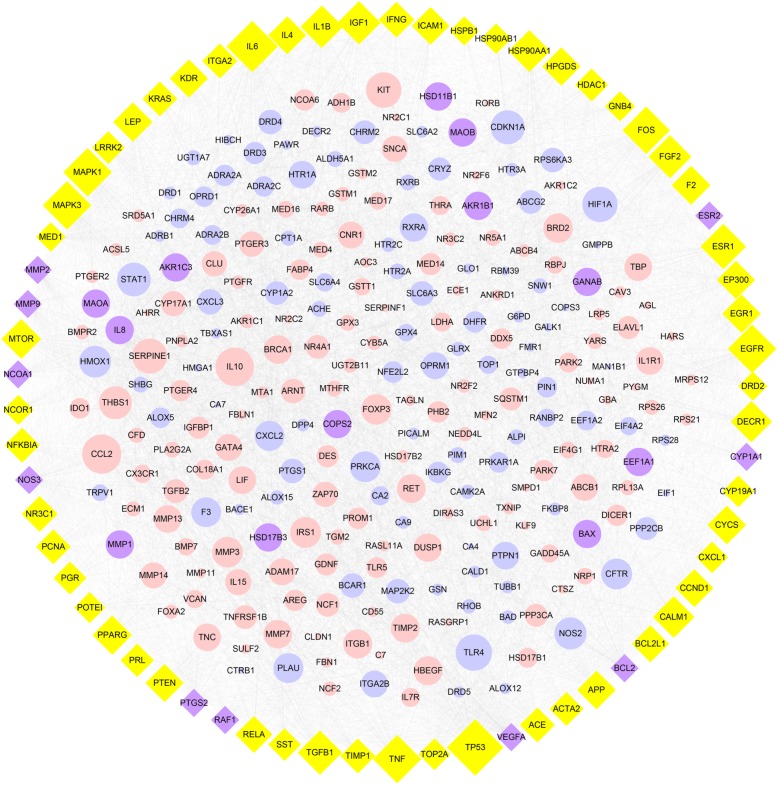
The core protein–protein interaction network. Pink circles, blue circles, Purple circles and yellow diamonds showed for the targets of disease, targets of compounds, 22 common targets, and 66 candidate targets, respectively. Purple diamonds represented the intersection between 66 candidate targets and 22 common targets. The size of circles and diamonds indicated nodes degree value.

By integrating data from disease databases, 401 EMS-related targets were acquired (**Supplementary Table S2**). The CT–DT network consisted of 592 nodes and 6166 edges. Targets of compounds were mapped to the EMS targets to obtain 22 common targets (**Supplementary Table S3**). Then a CPPI network including 22 targets and their first neighbors was extracted from the CT–DT network. The CPPI network comprised 315 nodes and 4703 edges (**Figure 3**). Subsequently, the average values of “Degree” and “Betweenness” for nodes were 29.8603 and 0.0039 in the CPPI network. The 66 nodes with “Degree” ≥ 29.8603 and “Betweenness” ≥ 0.0039 were chosen as the key targets (**Supplementary Table S4**). Interestingly, the 22 common targets were not completely contained in 66 key targets.

### Pathway Enrichment Analysis for Key Targets

**FIGURE 4:**
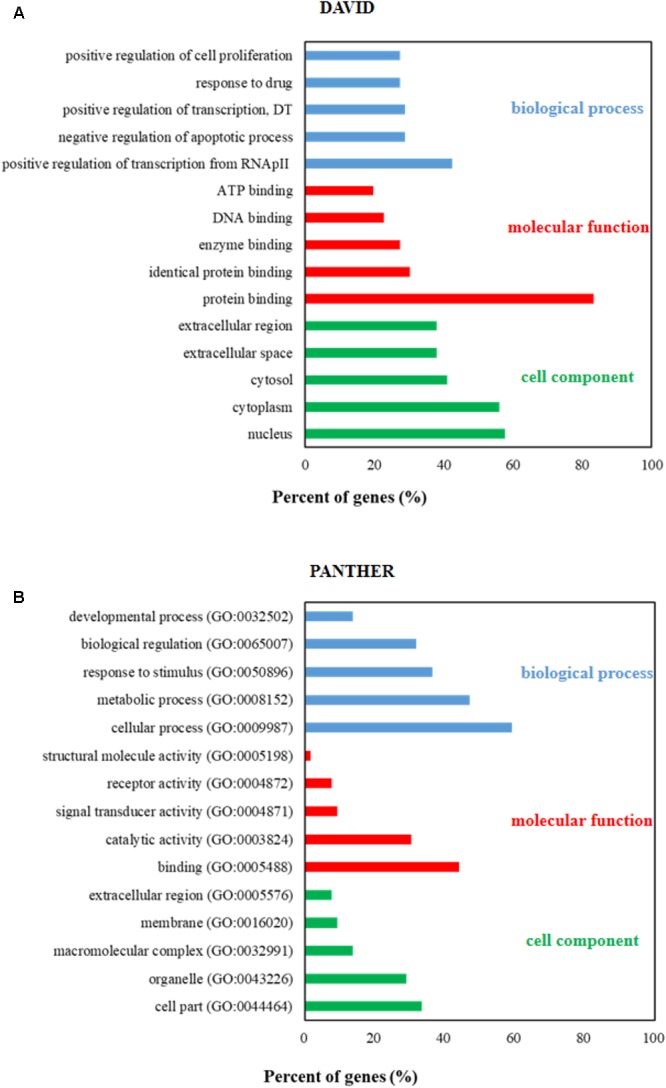
Gene ontology (GO) analysis of candidate targets. GO enrichment analysis in DAVID **(A)** and PANTHER **(B)** database showed the five remarkably enriched items in the biological processes, cell component, and molecular function. RNApII, RNA polymerase II promoter; DT, DNA-templated.

In order to further study the molecular mechanism of JFS on EMS, GO analysis and pathway enrichment of the 66 candidate targets were performed with KEGG and PANTHER database. The results of GO analysis were described by biological process (BP), cell component (CC), and molecular function (MF) terms. In KEGG database, 340 of 417 BPs, 34 of 42 CCs, and 60 of 76 MFs enriched for these targets were recognized as *P* < 0.05. Twelve BPs, six CCs, and five MFs were enriched from PANTHER database. An overview of the GO analysis was explored with top 5 remarkably enriched terms in the BP, CC, and MF categories (**Figure 4**). According to the results of pathway enrichment, 115 and 76 target-related pathways have been found in KEGG (**Supplementary Table S5**) and PANTHER database (**Supplementary Table S6**). Subsequently, remarkable 12 pathways were presented including MMP/TIMP (**Table 2**).

**Table 2:** Pathway including MMP-2, MMP-9, and TIMP-1

Pathway name	Gene name
Pathways in cancer	MMP-2, MMP-9
Proteoglycans in cancer	MMP-2, MMP-9
Estrogen signaling pathway	MMP-2, MMP-9
GnRH signaling pathway	MMP-2
Bladder cancer	MMP-2, MMP-9
Hepatitis B	MMP-9
TNF signaling pathway	MMP-9
Transcriptional misregulation in cancer	MMP-9
MicroRNAs in cancer	MMP-9
HIF-1 signaling pathway	TIMP-1
CCKR signaling map	MMP-9
Plasminogen activating cascade	MMP-9

### *Jiawei Foshou San* Inhibited the Volume of Ectopic Endometrium

**FIGURE 5:**
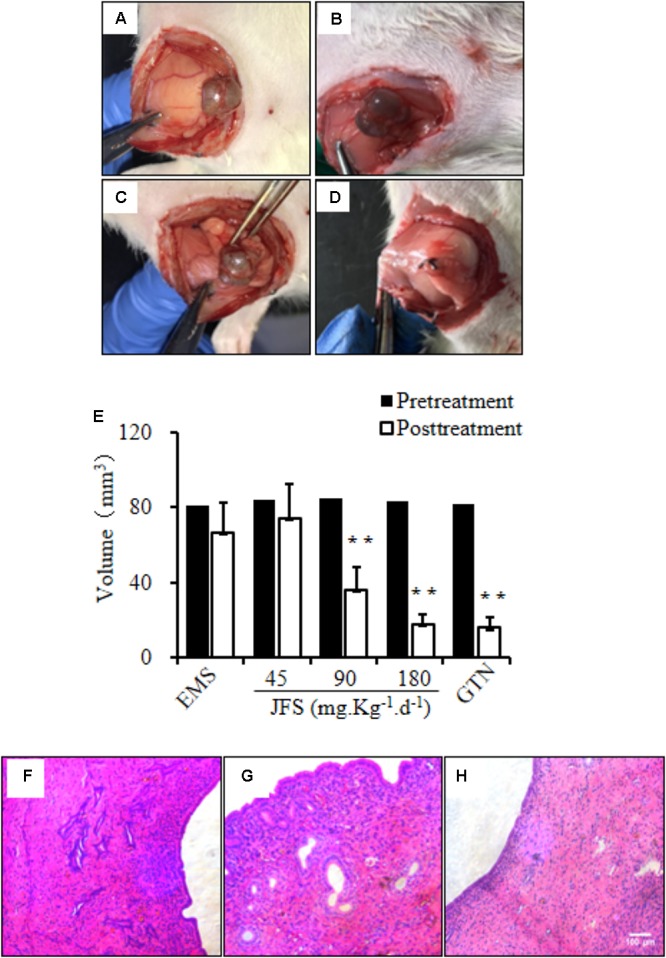
Regulation of JFS on volume and morphology of ectopic endometrium. The ectopic endometrium were observed before and after administration for 28 days in EMS group **(A,B)** and 180 mg.kg^-1^.d^-1^ JFS **(C,D)**. **(E)** The volume of ectopic endometrium were detected by Vernier caliper in rat endometriosis model. The morphological change of structure were found in eutopic endometrium in control **(F)**, ectopic endometrium in EMS **(G)**, and 180 mg.kg^-1^.d^-1^ JFS **(H)** in H&E staining. ^∗∗^*P* < 0.01 to pretreatment. Columns, mean (*n* = 6). Bars, SD. Magnification, ×100. EMS, endometriosis; JFS, *Jiawei Foshou San*; GTN, gestrinone.

After continuous gavage for 28 days, the volume of ectopic endometrium were evaluated and compared with pretreatment. There were no significantly varieties observed in EMS and JFS 45 mg.kg^-1^.d^-1^ groups. Using 90 or 180 mg.kg^-1^.d^-1^ JFS, the transplants became smaller, less adhesion and blood vessels outside, lower height of effusion. The volume of ectopic endometrium were 36.32 ± 11.78 and 17.90 ± 5.17 mm^3^, significantly reduced by 48.68 ± 12.19 and 65.29 ± 9.15 mm^3^, respectively (*P* < 0.01). Gestrinone diminished the volume of ectopic endometrium tissue from 81.92 ± 19.20 to 16.01 ± 5.53 mm^3^ (*P* < 0.01) (**Table 3** and **Figures 5A–E**). This suggests that JFS restrained the growth of ectopic endometrium in a dose-dependent manner.

**Table 3:** Effect of JFS on ectopic endometrium volume (*n* = 6).

Groups	Doses	Volume(mm^3^)		Volume changes (mm^3^)
		Pretreatment	Post-treatment	
EMS	-	80.94 ± 18.97	66.55 ± 16.01	14.39 ± 6.31
JFS	45 mg.kg^-1^.d^-1^	84.44 ± 17.03	74.54 ± 17.88	9.90 ± 2.48
	90 mg.kg^-1^.d^-1^	85.00 ± 18.42	36.32 ± 11.78^∗∗^	48.68 ± 12.19
	180 mg.kg^-1^.d^-1^	83.19 ± 12.27	17.90 ± 5.17^∗∗^	65.29 ± 9.15
GTN	50 mg.kg^-1^.d^-1^	81.92 ± 19.20	16.01 ± 5.53^∗∗^	65.91 ± 14.34

*JFS, Jiawei Foshou San; GTN, gestrinone. Values were mean ± SD. ^∗^ P < 0.05, ^∗∗^P < 0.01 vs. pretreatment.*

### Improvement of Ectopic Endometrium Morphology by *Jiawei Foshou San*

In H&E staining, the ectopic endometrium in EMS group had a similar structure with eutopic endometrium in control group. The ectopic endometrium were constituted with endometrial glandular epithelial cell, endometrial stromal cell, and fibrous connective tissue. After gavage of 90 and 180 mg.kg^-1^.d^-1^ JFS, the amelioration of ectopic endometrium structure were found, such as thinner ectopic endometrium, looser cell arrangement, less pseudoglandular, decreased blood vessels and inflammatory cells (**Figures 5F–H**).

### *Jiawei Foshou San* Suppressed Invasion and Metastasis

**FIGURE 6:**
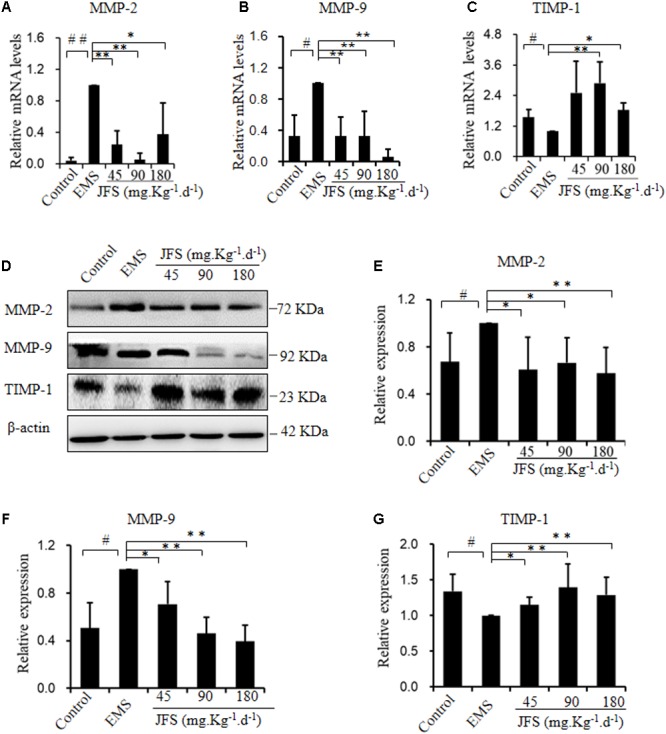
Effect of JFS on invasion and metastasis. **(A–C)** The mRNA levels of MMP-2, MMP-9, and TIMP-1 were detected by qPCR in different groups. **(D–G)** The protein levels of MMP-2, MMP-9, and TIMP-1 were detected by western blotting, and the ratio of MMP-2, MMP-9, and TIMP-1 with β-actin were shown. ^#^*P* < 0.05 to control, ^##^*P* < 0.01 to control, ^∗^*P* < 0.05 to EMS, ^∗∗^*P* < 0.01 to EMS. Columns, mean (*n* = 3). Bars, SD. EMS, endometriosis; JFS, *Jiawei Foshou San*.

MMP-2, MMP-9, and TIMP-1 are considered as the important roles in invasion and metastasis (Li et al., 2017). The gene and protein expression of MMP-2, MMP-9, and TIMP-1 were tested in our study. The mRNA levels of MMP-2 and MMP-9 significantly rose in EMS group (*P* < 0.05), while the mRNA level of TIMP-1 declined (*P* < 0.05). On the contrary, JFS obviously inhibited the gene expression of MMP-2 (*P* < 0.05) and MMP-9 (*P* < 0.01) compared with EMS group. Meanwhile the mRNA level of TIMP-1 was significantly upregulated in 90 and 180, not 45 mg.kg^-1^.d^-1^ JFS group, compared with EMS group (*P* < 0.05) (**Figures 6A–C**). Remarkably higher protein levels of MMP-2 and MMP-9 were founded in EMS group, with lower protein level of TIMP-1 than those in control group (*P* < 0.05). While treated with JFS, MMP-2, MMP-9 protein were decreased significantly in a dose-dependent manner (*P* < 0.05). The protein level of TIMP-1 was increased in three JFS groups vs. EMS group (*P* < 0.05) (**Figures 6D–G**). This suggests that the abatement of invasion and metastasis by JFS were connected with increasing MMP-2, MMP-9, and decreasing TIMP-1.

### Reverse of Epithelial–Mesenchymal Transition by *Jiawei Foshou San*

**FIGURE 7:**
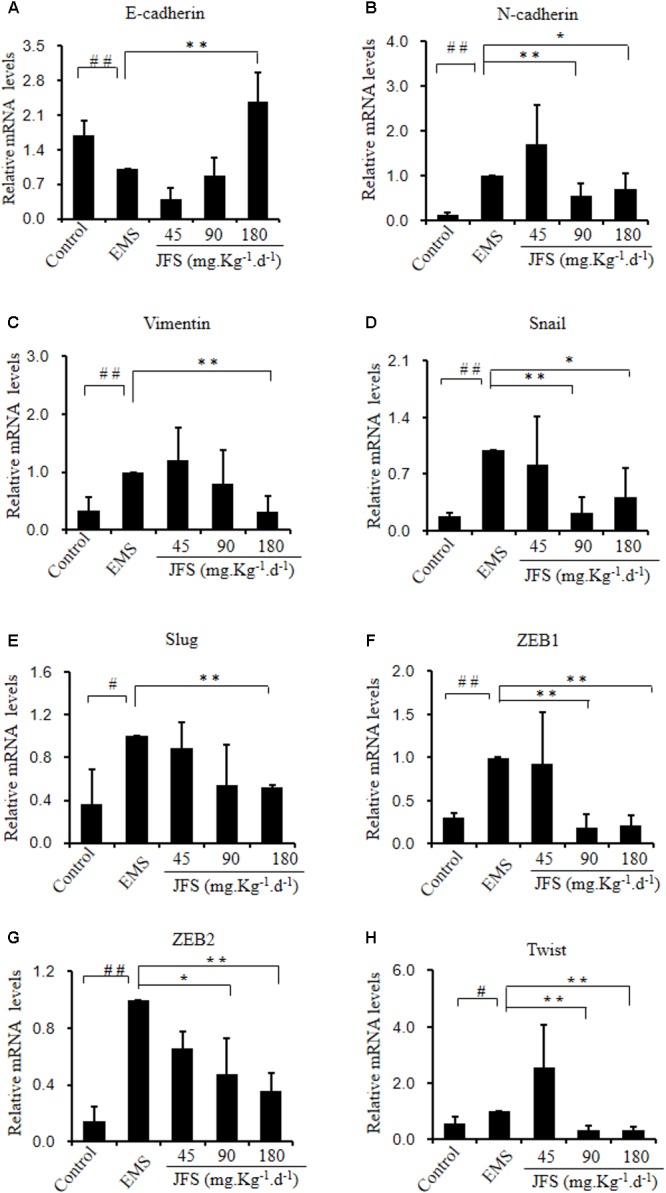
Gene expression of epithelial–mesenchymal transition adjusted by JFS. **(A)** The epithelial marker E-cadherin mRNA level was measured by qPCR. **(B–H)** Mesenchymal genes expression of N-cadherin, Vimentin, Snail, Slug, ZEB1, ZEB2, and Twist using qPCR. ^#^*P* < 0.05 to control, ^##^*P* < 0.01 to control, ^∗^*P* < 0.05 to EMS, ^∗∗^*P* < 0.01 to EMS. Columns, mean (*n* = 3). Bars, SD. EMS, endometriosis; JFS, *Jiawei Foshou San*.

**FIGURE 8:**
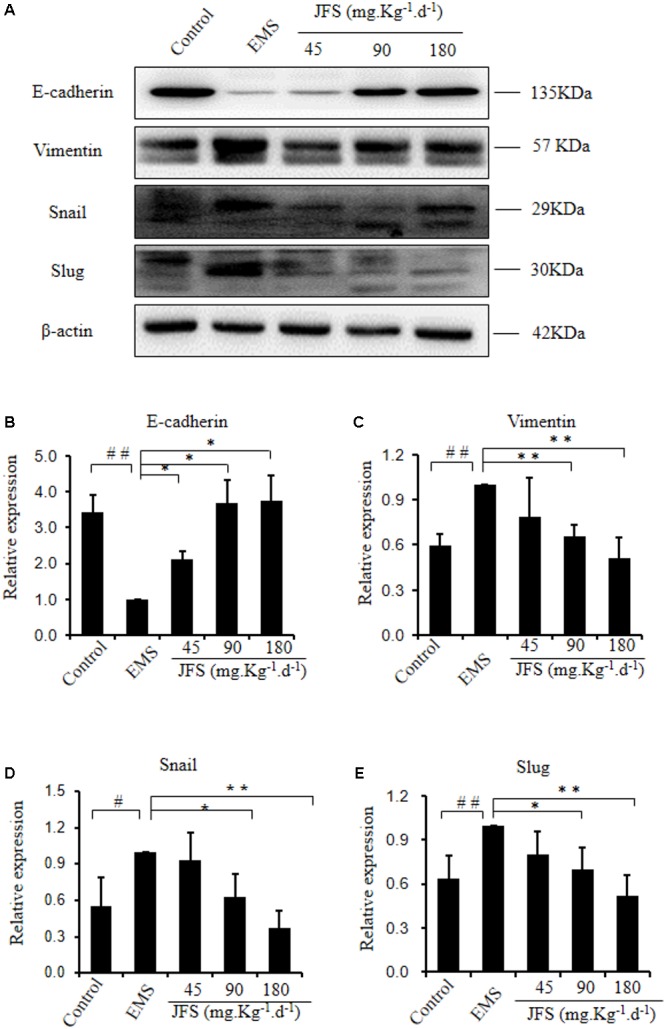
Modification of epithelial–mesenchymal transition protein treating with JFS. **(A–E)** The protein levels of E-cadherin, Vimentin, Snail, and Slug were detected by western blotting, and the ratio of E-cadherin, Vimentin, Snail, and Slug with β-actin were shown. ^#^*P* < 0.05 to control, ^##^*P* < 0.01 to control, ^∗^*P* < 0.05 to EMS, ^∗∗^*P* < 0.01 to EMS. Columns, mean (*n* = 3). Bars, SD. EMS, endometriosis; JFS, *Jiawei Foshou San*.

Epithelial–mesenchymal transition is related with invasion and metastasis (Zheng et al., 2015). E-cadherin gene expressed significantly lower in EMS group vs. control group (*P* < 0.01). In contrast, N-cadherin, Vimentin, Snail, Slug, ZEB1, ZEB2, and Twist mRNA were significantly higher in EMS group than those in control group (*P* < 0.05). Since administration of JFS, the transition from epithelial phenotype to mesenchymal phenotype were reversed, for instance, upregulated mRNA level of E-cadherin, downregulated mRNA levels of N-cadherin, Vimentin, Snail, Slug, ZEB1, ZEB2, Twist compared with EMS group (*P* < 0.05) (**Figure 7**). Remarkably higher protein levels of Vimentin, Snail, and Slug were founded in EMS group, with lower protein level of E-cadherin than those in control group (*P* < 0.05). While using JFS, Vimentin, Snail, and Slug protein were decreased significantly in a dose-dependent manner (*P* < 0.05). The protein level of E-cadherin was increased in three JFS groups vs. EMS group (*P* < 0.05) (**Figure 8**).

## Discussion

According to the reflux theory of menstruation, invasion and metastasis of ectopic endometrium is the vital step in EMS, especially following with degradation of extracellular matrix. MMP-2, MMP-9, and TIMP-1 are the important factors in invasion and metastasis, and balance between them regulate degradation of extracellular matrix. In previous study, MMP-2 and MMP-9 increased, meanwhile TIMP-1 decreased in EMS (Huang et al., 2015; Yi et al., 2015; Jana et al., 2016; Szymanowski et al., 2016). The therapy, through suppressing MMP-2 and MMP-9, promoting TIMP-1, have been reported as the effective methods on EMS (Huang et al., 2015; Li et al., 2016; Kim et al., 2017). Consistently, in our experiment, JFS inhibited the growth of ectopic endometrium and recurred the pathological changes. It might be related with the regulation of MMP-2, MMP-9, and TIMP-1 to suppress invasion and metastasis. It is worthwhile to explore role of other MMP or TIMP, for example, MMP-1, MMP-7, TIMP-2 in EMS, and the effect of JFS on them in future.

Using network pharmacological analysis, the kernel targets were collected from the CPPI network for pathway enrichment. Interestingly, we found that there were some pathways related to MMP or TIMP, including estrogen, GnRH and TNF pathways. Estrogen, GnRH, and TNF are the pivotal mediators of endometrial homeostasis (Granese et al., 2015; Leavy, 2015; Simmen and Kelley, 2016; Tosti et al., 2017). Furthermore, MMP and TIMP are regulated by these three pathways (Raga et al., 1999; Voloshenyuk and Gardner, 2010; Yang et al., 2012). In our previous study, JFS suppressed GnRH, estrogen, and TNF (Tang et al., 2014). These might be the reasons of regulating MMP/TIMP balance by JFS.

Epithelial–mesenchymal transition presents the features, loss of polarity, and cellular adhesion in epithelial cells, convert into mesenchymal phenotype (Thiery et al., 2009). During this process, epithelial surface markers (e.g., E-cadherin, keratin) lose, mesenchymal markers (e.g., Vimentin, N-cadherin) express, and more migration and invasion subsequently emerge. Snail, Slug, ZEB1, ZEB2, and Twist, as the transcription factors, induce epithelial–mesenchymal transition through depressing E-cadherin (Cano et al., 2000; Zhang et al., 2006; Chen et al., 2010). Recently, lower E-cadherin, and higher Vimentin, N-cadherin, Snail, Slug, ZEB1, ZEB2, and Twist have been demonstrated in EMS (Furuya et al., 2017; Chatterjee et al., 2018; Wang et al., 2018). Interestingly, epithelial–mesenchymal transition has been inhibited with decline of MMP expression (Wu et al., 2018). We also found that E-cadherin increased, and Vimentin, N-cadherin, Snail, Slug, ZEB1, ZEB2, and Twist decreased. JFS restrained epithelial–mesenchymal transition, at the same time suppressed MMPs and promoted TIMP-1. These consistent data indicated a potential mechanism of increasing invasion and metastasis by JFS through epithelial–mesenchymal transition.

*Foshou San* formula is composed of *Ligusticum chuanxiong* Hort and *Angelica sinensis.* Ligustrazine from *L. chuanxiong* Hort, ferulic acid from *A. sinensis*, and tetrahydropalmatine are mixed to JFS with the certain proportion. Firstly, in previous study, ligustrazine has the anti-metastatic effects through decreasing MMP-2, MMP-9, MMP-3, MMP-13, increasing TIMP-1, TIMP-2 (Liang et al., 2014; Jiang et al., 2017; Fang et al., 2018). But up-regulating expression of MMP-2 and MMP-9 is found in bone marrow mesenchymal stem cells by ligustrazine (Wang et al., 2016). These results suggest that ligustrazine might have the different influence on metastasis in different diseases. Tetramethylpyrazine also inhibits epithelial–mesenchymal transition progression (Luan et al., 2016). Secondly, treated with ferulic acid alone or combination with other drugs, its role in suppression of metastatic potential are regulated by the reversal of epithelial–mesenchymal transition (Wei et al., 2015; Zhang et al., 2016). Thirdly, tetrahydropalmatine had a negative effect against invasion in cancer (Yodkeeree et al., 2013). While levo-tetrahydropalmatine attenuates blood–brain barrier injury and brain edema, but inhibits MMP-2/9 (Mao et al., 2015). In our study, JFS restrained metastasis through accumulating TIMP-1 and attenuating MMP-2, MMP-9. In addition, epithelial–mesenchymal transition were recovered. With all above confused results, the mechanism of JFS on metastasis and epithelial–mesenchymal transition need for further investigation.

## Conclusion

In conclusion, these results showed that CPPI network was established through analysis of JFS targets with EMS targets. Then 66 kernel targets were selected for pathway enrichment. In EMS model, JFS was able to inhibit growth and pathological change. Furthermore, the modification of MMP/TIMP balance and down-regulation of epithelial–mesenchymal transition might be the potential mechanisms for JFS on EMS. These findings provide logical support for further evaluation of JFS.

## Author Contributions

YC and JW performed the major research in equal contribution. YZ, WS, ZL, QW, and XX provided the technical support. CL contributed to final approval of the version to be published. PL designed the study and revised the manuscript.

## Conflict of Interest Statement

The authors declare that the research was conducted in the absence of any commercial or financial relationships that could be construed as a potential conflict of interest.

## Footnotes

1 http://www.megabionet.Org/tcmid/search/

2 http://lsp.nwu.edu.cn/tcmsp.php, updated on May 31, 2014

3 http://sea.bkslab.org/

4 https://omim.org/, updated on Oct 31, 2017

5 http://www.disgenet.org/, last updated May 28, 2017

6 https://www.ncbi.nlm.nih.gov/geo/

7 http://www.uniprot.org/, last modified June 22, 2017

8 https://string-db.org/

9 https://david.ncifcrf.gov/, version 6.8

10 http://pantherdb.org, version 13.1

## Supplementary Material

TABLE S1 Detail and interaction information of targets for JFS compounds.

TABLE S2 EMS-related targets and interaction information with JFS-related targets.

TABLE S3 22 common targets obtained from JFS-related targets and EMS-related targets.

TABLE S4 66 key targets extracted from a core protein–protein interaction network.

TABLE S5 GO analysis and KEGG pathway enrichment of 66 key targets from DAVID database.

TABLE S6 GO analysis and pathway analysis of 66 key targets from PANTHER database.

## Abbreviations

CPPI: core protein–protein interaction
CT–DT: compound target–disease target
EMS: endometriosis
JFS: *Jiawei Foshou San*
TCM: traditional Chinese medicine
